# TADOSS: computational estimation of tandem domain swap stability

**DOI:** 10.1093/bioinformatics/bty974

**Published:** 2018-11-30

**Authors:** Aleix Lafita, Pengfei Tian, Robert B Best, Alex Bateman

**Affiliations:** 1 European Molecular Biology Laboratory, European Bioinformatics Institute, Wellcome Genome Campus, Hinxton, Cambridge, UK; 2 Laboratory of Chemical Physics, National Institute of Diabetes and Digestive and Kidney Diseases, National Institutes of Health, Bethesda, MD, USA

## Abstract

**Summary:**

Proteins with highly similar tandem domains have shown an increased propensity for misfolding and aggregation. Several molecular explanations have been put forward, such as swapping of adjacent domains, but there is a lack of computational tools to systematically analyze them. We present the TAndem DOmain Swap Stability predictor (TADOSS), a method to computationally estimate the stability of tandem domain-swapped conformations from the structures of single domains, based on previous coarse-grained simulation studies. The tool is able to discriminate domains susceptible to domain swapping and to identify structural regions with high propensity to form hinge loops. TADOSS is a scalable method and suitable for large scale analyses.

**Availability and implementation:**

Source code and documentation are freely available under an MIT license on GitHub at https://github.com/lafita/tadoss.

**Supplementary information:**

Supplementary data are available at *Bioinformatics* online.

## 1 Introduction

Protein misfolding and aggregation is a major problem for cells and organisms, and the cause of severe human diseases like Alzheimer’s. Recent studies have shown an increased propensity of aggregation in proteins containing identical domains in tandem (Borgia *et al.*, 2015; Wright *et al.*, 2005). One of the misfolded conformations identified in these experiments are domain swaps, i.e. part of the structure of a domain folds into its adjacent domain. Domain swapping has been associated with protein aggregation (Rousseau *et al.*, 2012), so its computational prediction is of widespread biomedical and biotechnological interest.

A recent study by Tian and Best (2016) demonstrated using coarse-grained simulations of tandem pairs of identical domains that the difference in stability between the native and the domain-swapped conformations correlates with the swapping propensity. They also described an alchemical approach (i.e. simplified model) to estimate the free energy difference of the two conformations that can be generally used to predict domain swapping. Here we present an improved and fully automated version of the method, named TAndem DOmain Swap Stability predictor (TADOSS), which can be used to systematically find domains susceptible to domain swapping and identify the regions of the structure with the highest propensity to form hinge loops.

## 2 Description

**Fig. 1.**
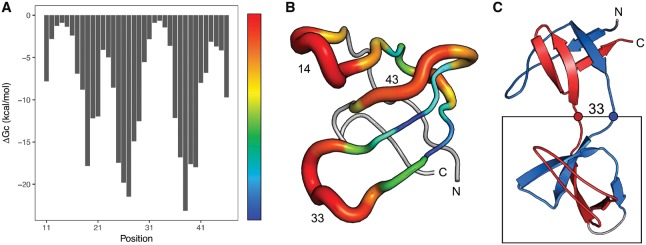
Alchemical free energy of forming a hinge loop ($\Delta G_{C}$) centered at every residue position in an SH3 domain (ECOD: e1shgA1). Energies can be visualized as a profile along the domain sequence (**A**) or mapped in 3D to the domain structure (**B**). Higher (more positive) free energy differences (red) indicate regions of the structure more likely to form hinge loops. For the SH3 domain, the alchemical free energy of joining the termini ($\Delta G_{J}$) is −6.1 kcal/mol. The most stable domain swap conformation is predicted to be forming a hinge loop centered at Position 33, with a total ΔΔG of −6.8 kcal/mol. The corresponding tandem domain-swapped structure extracted from the simulations by Tian and Best (2016) is also shown (**C**)

As described originally by Tian and Best (2016), the total free energy difference between the native and swapped conformations can be split into the energy of joining the N- and C- termini of the domain ($\Delta G_{J}$) and the energy of cutting the domain ($\Delta G_{C}$), i.e. forming a hinge loop between the swapped domains. TADOSS systematically evaluates all possible cut positions in the domain and calculates the free energy contribution of forming a hinge loop of at least three residues. The profile of $\Delta G_{C}$ is valuable to identify the regions of the domain that are more susceptible to form hinge loops in the tandem domain swaps, as shown in Figure 1.

The length of the linker between adjacent domains also plays an important role in the stability of tandem domain swaps. Longer linkers allow the connection of the N- and C-termini of the domain, thereby increasing the $\Delta G_{J}$. To account for this effect, we have introduced an optional parameter in TADOSS to reduce the effective distance between the termini of the domain proportional to the length of the linker.

Finally, the total free energy difference ΔΔG of a tandem domain swap is obtained by summing up the free energy differences for cutting ($\Delta G_{C}$) and joining ($\Delta G_{J}$) the domain. The most susceptible domain swaps are those with the maximum ΔΔG (most positive). More information about the energetic model and its parameters can be found in the supplementary materials.

## 3 Results

The alchemical free energy difference from TADOSS correlates well with the free energies obtained in the simulations by Tian and Best (2016), although the scale differs by a factor of two approximately (Supplementary Fig. S1). We also find an agreement in the effect of the linker length between the simulated and alchemical free energy differences (Supplementary Fig. S2).

Using the free energy profile from TADOSS, it is possible to reproduce experimental ΔG measurements and identify with good accuracy the folding units of a DHFR domain characterized by Iwakura *et al.* (2000) (Supplementary Fig. S3). Furthermore, hinge loops of experimentally determined domain swap dimers presented by Ding *et al.* (2006) correspond to maximums of the $\Delta G_{C}$ profile, as expected (Supplementary Fig. S4).

We also provide a dataset of alchemical ΔΔG estimations for T-group (topology) representatives in the ECOD database (Cheng *et al.*, 2014). We find a significant proportion of domains susceptible to tandem domain swapping (Supplementary Fig. S5).

## 4 Implementation

TADOSS is written in Python and bundled in a Bash script with a simple interface to the user. The program takes the structure of a protein domain as a PDB file and generates output files with the alchemical free energy differences. The method requires BioPython (Cock *et al.*, 2009) to parse and manipulate the domain structures and either GROMACS (Abraham *et al.*, 2015) or Reduce (Word *et al.*, 1999) to add the hydrogens.

The structure of the input domain is represented using a coarse-grained structure-based (Gō-like) model, as described by Karanicolas and Brooks (2002). Native interactions in the structure are attractive and the relative contact energies are set according to the Miyazawa-Jernigan matrix (Miyazawa and Jernigan, 1996).

The running time for an example domain of 159 residues on a MacBook Pro 2.9 GHz Intel Core i5 with 16 GB RAM is about 4 s. The method scales quadratically with the number of residues in the input domain structure (Supplementary Fig. S6).

## Funding

This work was supported by the intramural research program of the National Institute of Diabetes and Digestive and Kidney Diseases (grant number ZIA DK075104-06) of the National Institutes of Health to P.T. and R.B.B. This work was funded by the European Molecular Biology Laboratory.

*Conflict of Interest*: none declared.

## Supplementary Material

bty974_Supplementary_Data
