# Supplementary material for: TADOSS: computational estimation of tandem domain swap stability
Source: Bioinformatics. 2018 Nov 30;35(14):2507–8. doi: 10.1093/bioinformatics/bty974 (PMC6612889; doi:10.1093/bioinformatics/bty974)
Supplement: bty974_Supplementary_Data [file bty974_supplementary_data.zip › bty974-Suppl_data/Supplementary_Data_energetic_model.pdf]

Description of the energetic model

The stability of a domain can be described by the difference of the absolute free energy between the folded and unfolded states.

$$\Delta G = G_F - G_U \tag{1}$$

The free energy difference can be further split into the change in energetics (E) and entropy (S) between the states.

$$\Delta G = \Delta E - T\Delta S \tag{2}$$

We define the relative stability of a native domain (WT) to its domain swapped version (SW) as the difference in their free energy differences.

$$\Delta\Delta G = \Delta G_{WT} - \Delta G_{SW} \tag{3}$$

Since the native and the swapped domains have the same amino acid sequences, we assume their unfolded states to have the same absolute free energy (G_U). In that case, the change in stability only depends on the absolute free energy of their folded states.

$$\Delta\Delta G = G_{WT} - G_{SW} \tag{4}$$

$\Delta\Delta G$ is negative if the native state is more stable relative to the tandem swapped state.

Comparing the native and the domain swapped structures we can observe that the majority of the native contacts are conserved, so their energetic and entropic contributions to the free energy will be the same and hence their $\Delta\Delta G$ will be zero. Therefore, we can estimate the $\Delta\Delta G$ (defined as $\Delta\Delta G_{alchemical}$) using the contributions of 1) the sum of the energy ϵ of each native contact disrupted (broken) C:

$$\Delta\Delta E_{alchemical} = \sum_{c}^{C} \epsilon_c \tag{5}$$

and 2) the total entropy loss in the native conformation, calculated from the entropic gain of unfolding one residue δs by the total number of residues unfolded R_U in the domain swap:

$$\Delta\Delta S_{alchemical} = R_U\ (-\delta s) \tag{6}$$

Therefore, the energetic component will have a negative contribution to to total free energy (contacts are formed going from the swap to the native conformation), while the entropic component will have a positive contribution (unfolded residues in the swap become folded in the native structure). The alchemical $\Delta\Delta G$ can be expressed as:

$$\Delta\Delta G_{alchemical} = \sum_{c}^{C} \epsilon_c - T \cdot R_U \ (-\delta s) \tag{7}$$

We can further distinguish two sets of native contacts that will be disrupted in the domain swapped state: 1) contacts at the termini, disrupted when residues are peeled off the the N- and C-terminal when they are covalently joined, and 2) contacts at the hinge loop, disrupted when a region of the domain is extended to form a linker between the two domains. For consistency with the original method description, we define their alchemical free energy contributions as following:

$$\Delta\Delta G_{alchemical} = \Delta G_J + \Delta G_C \tag{8}$$

The topological requirement for joining the termini is that the linear distance between residues at position i and j is shorter than the effective length contributed by the peeled off residues:

$$d(i,j) < (i + L - j - M - L_l) \ r_0 \tag{9}$$

Where L is the length of the protein (number of residues), L_l is the interdomain linker length and M is a penalty for the need of a loop when the termini point in opposite directions, defined as:

$$M = 6 \ \sin(\frac{\theta}{2}) \tag{10}$$

In the above expression, θ is the angle between the chain directions at the N and C-terminus. The N-terminal chain direction is defined as the vector between residues i and $i+4$ and the C-terminal direction as the vector between residues $j-4$ and j.

Now, considering C_J to be the contacts of residues $\{1..i\}$ and $\{j..L\}$, the ΔG_J is set to the maximum (most positive value) of all possible i and j that fulfill the topological requirement:

$$\Delta G_J = \max_{i,j \in \{1..10\}} \left\{ \sum_{c}^{C_J} \epsilon_c + T(i + L - j) \ \delta s \right\} \tag{11}$$

On the other hand, considering C_C to be the contacts of the residues centered at the cut position forming the hinge loop between domains, the ΔG_C is set to the maximum (most positive value) of all possible hinge loop lengths h between the minimum L_h and maximum (set to 8 by default).

$$\Delta G_C = \min_{h \in \{L_h..8\}} \left\{ \sum_{c}^{C_C} \epsilon_c + T \cdot h \cdot \delta s \right\} \tag{12}$$

Model parameters

- Entropy gain of unfolding: $\delta s = 0.0054$ kcal/mol $\cdot$ K $\cdot$ residue
- Temperature: $T = 350\ K$
- Minimum length of the hinge loop: $L_h = 3$ residues
- Length of the inter-domain linker: $L_l = 0$ residues
- Average length contribution of a residue: $r_0 = 3.5$ Å
