# Supplementary material for: TADOSS: computational estimation of tandem domain swap stability
Source: Bioinformatics. 2018 Nov 30;35(14):2507–8. doi: 10.1093/bioinformatics/bty974 (PMC6612889; doi:10.1093/bioinformatics/bty974)
Supplement: bty974_Supplementary_Data [file bty974_supplementary_data.zip › bty974-Suppl_data/Supplementary_Data.pdf]

TADOSS: computational estimation of tandem domain swap stability

Supplementary figures

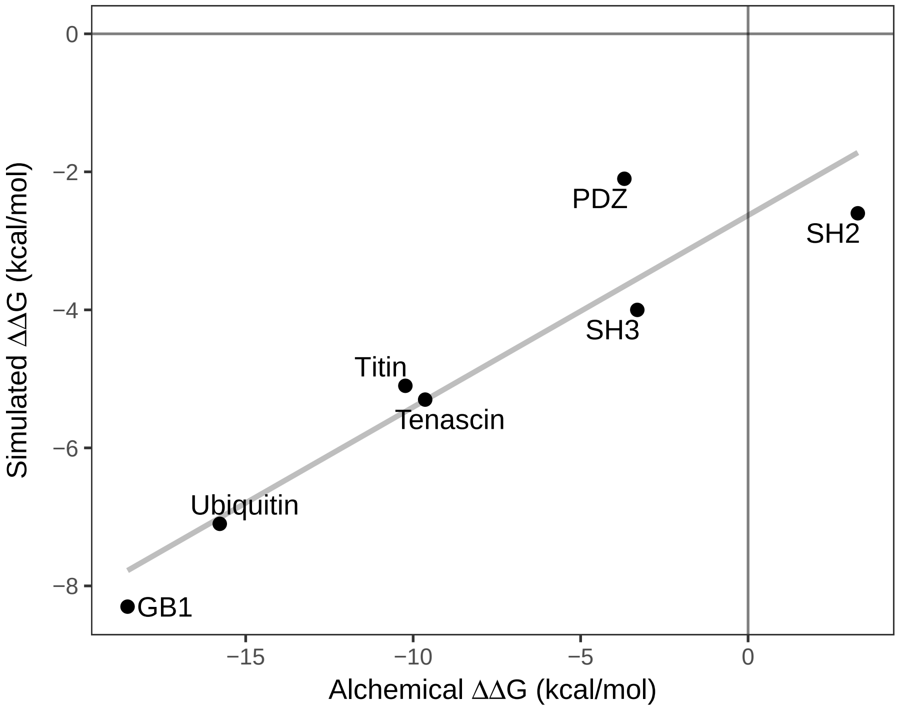

Figure S1: Correlation between the simulated and alchemical $\Delta\Delta G$ for the most stable domain-swapped misfold of each of the different domains analyzed by Tian and Best (2016): SH3 (1SHG), SH2 (1TZE), PDZ (2VWR), Tenascin (1TEN), Titin (1TIT), Ubiquitin (1UBQ), and GB1 (1GB1).

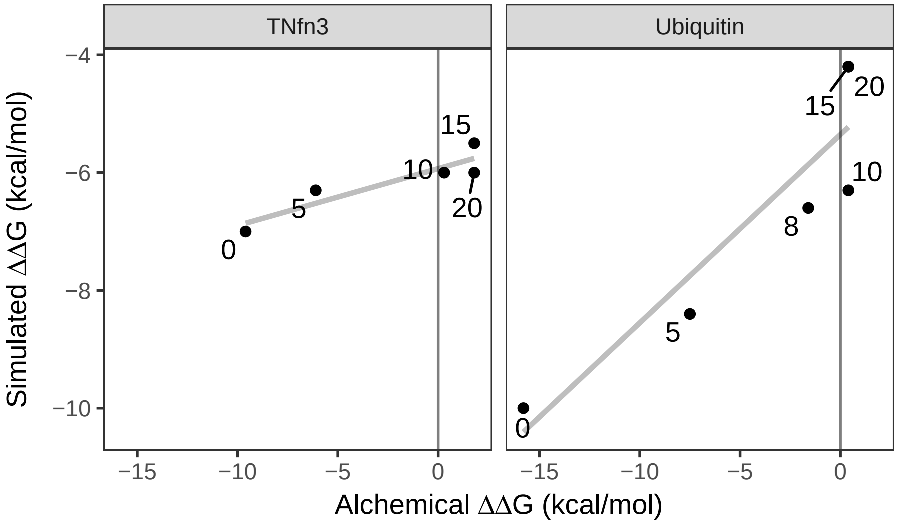

Figure S2: Correlation between the simulated and alchemical $\Delta\Delta G$ for different inter-domain linker lengths of a Fibronectin type III domain from Tenascin (TNFn3, 1TEN) and Ubiquitin domain (1UBQ).

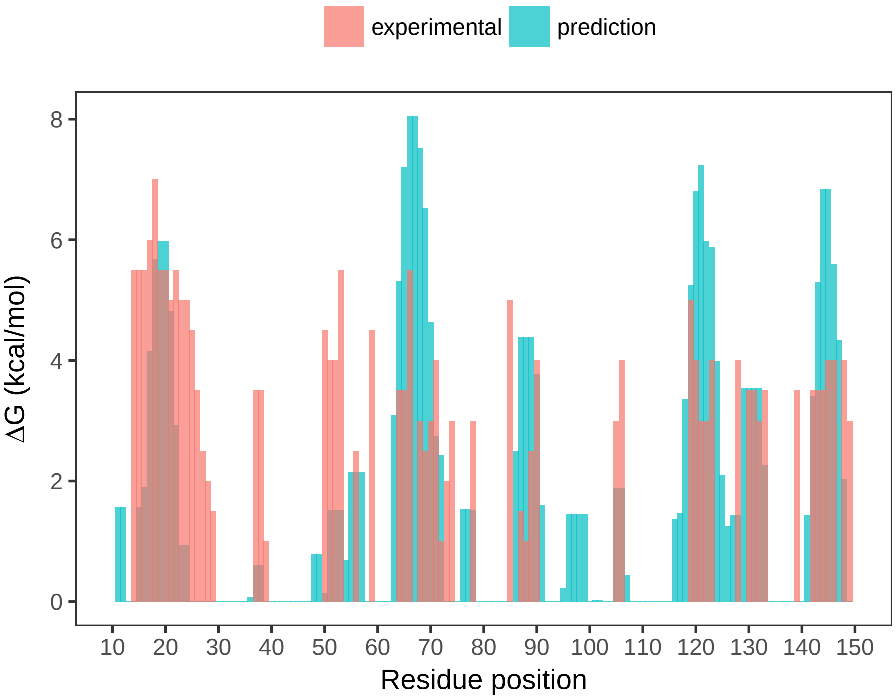

Figure S3: Comparison of the experimental unfolding ΔG of circular permuted versions of a DHFR domain (1RX4) measured by Iwakura *et al.* (2000) to the alchemical cut free energy (ΔG_C) prediction. Peaks on the ΔG profile indicate structural regions prone to circular permutations (the region that is cut to form the new N- and C-termini of the circular permutation). The experimental setup included a linker length of 5 residues, which reduced the alchemical ΔG_J of the domain to a negligible -0.1 kcal/mol, therefore making ΔG_C directly comparable to the experimental measurement. In the calculation of alchemical ΔG_C, a hinge loop of minimum length 0 is used to account for the prediction of circular permutations instead of domain swaps. In circular permutations the cut position is being disconnected, but it does not extend to form a hinge loop. Experimental ΔG is set to 0 in cases where the DHFR circular permutant constructs did not fold (regions that were further determined as folding units).

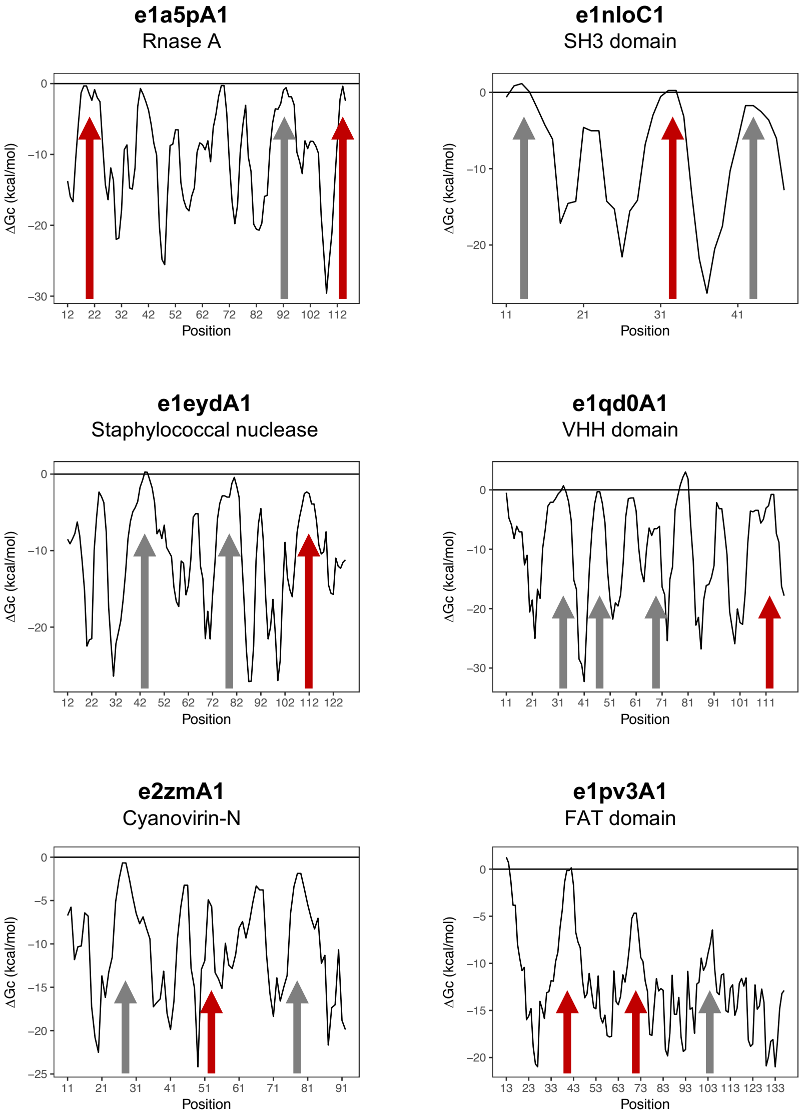

Figure S4: Detection of hinge loop regions in the domain examples presented by Ding *et al.* (2006). Although the experimentally observed domain swap dimers (red arrows) are not always predicted as the most probable hinge loop region by TADOSS (only 3 out of 6), they are always found in a maximum of the ΔGc profile. Additionally, TADOSS mostly agrees with the other hinge loop predictions by Ding *et al.* (grey arrows). Only ΔG_C is used in this analysis to predict domain swap dimers (no termini joining is required) instead of tandem domain swaps.

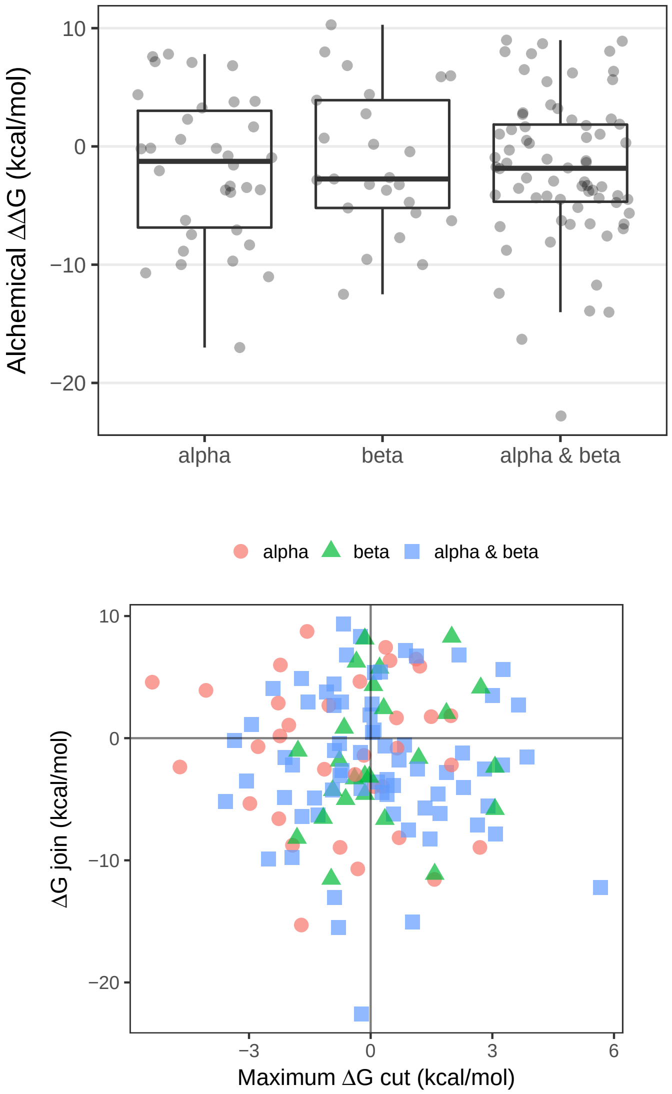

Figure S5: Alchemical $\Delta\Delta G$ (top), ΔG_C and ΔG_J (bottom) across manual representatives for each T-group (topology) of the ECOD database (Cheng *et al.*, 2014). Domains are split according to their secondary structure composition: alpha helical (alpha), beta sheets (beta), or part alpha helical and part beta sheet (alpha & beta). 38% of the domains have a positive alchemical $\Delta\Delta G$, with small differences among secondary structure content, of which 18% have both positive ΔG_C and ΔG_J. The raw data for this figure can be found in the GitHub repository: `https://github.com/lafita/tadoss/blob/master/census/ecod_topology_manual-reps.tsv`

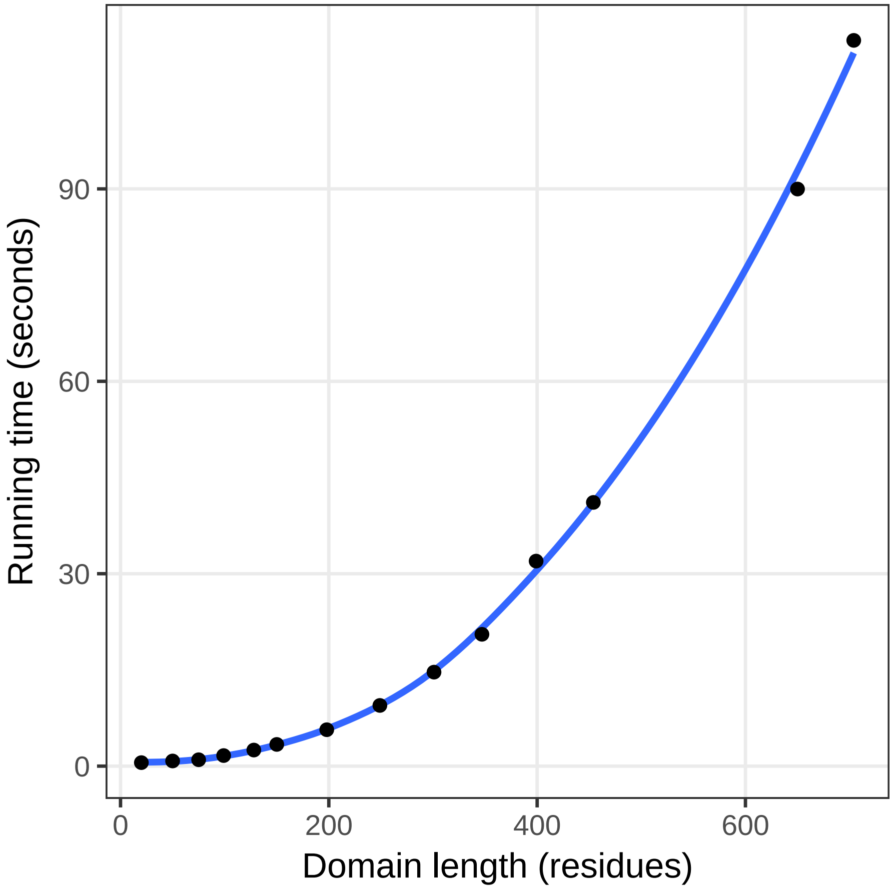

Figure S6: CPU time to calculate the alchemical $\Delta\Delta G$ with TADOSS as a function of the length of the input domain. Calculations performed on random ECOD domains of different lengths, sorted increasingly: e2bl6A2, e1nh2D2, e2egeA1, e3pv5B2, e1c20A1, e2b06A1, e3c6aA1, e4jkxA1, e4g3hA1, e1uqyA1, e4e4jA1, e1ua4A1, e1lkxA1, and e2iukA4. TADOSS was run on a MacBook Pro 2.9 GHz Intel Core i5 with 16 GB RAM. TADOSS running time is quadratic in the number of residues of the input structure. An domain of average length (100 residues) takes less than 2 seconds.

References

Cheng, H. *et al.* (2014). ECOD: An Evolutionary Classification of Protein Domains. *PLoS Computational Biology*, **10**(12).

Ding, F. *et al.* (2006). Topological determinants of protein domain swapping. *Structure*, **14**(1), 5–14.

Iwakura, M. *et al.* (2000). Systematic circular permutation of an entire protein reveals essential folding elements. *Nature Structural Biology*, **7**(7), 580–585.
